# Supplementary material for: Differential spatiotemporal development of Purkinje cell populations and cerebellum-dependent sensorimotor behaviors
Source: eLife. 2021 May 11;10:e63668. doi: 10.7554/eLife.63668 (PMC8195607; doi:10.7554/eLife.63668)
Supplement: Supplementary file 1. — Descriptive values are represented as mean ± SEM. p=postnatal, Z−=ZebrinII negative, Z+=ZebrinII positive, LIII = lobule III, LX = lobule X, LI-III = lobules I/II/III, LIX-X = lobules IX/X. [file elife-63668-supp1.docx]

**Supplementary File 1**

| **Related Figure** | **Test applied** | **Groups** | ***p*-value** | **Descriptive values** | **Number of cells / mice** |
| --- | --- | --- | --- | --- | --- |
| Figure1 C_1_  Simple spike rate (*in vivo*) | Two-way ANOVA multiple comparisons | P12-P17 Z– vs P12-P17 Z+  P18-P29 Z– vs P18-P29 Z+  P30-P59 Z– vs P30-P59 Z+  >P60 Z– vs >P60 Z+  >P60 Z– vs P12-17 Z–  >P60 Z– vs P18-29 Z–  >P60 Z– vs P30-P59 Z–  >P60 Z+ vs P12-17 Z+  >P60 Z+ vs P18-29 Z+  >P60 Z+ vs P30-P59 + | 0.013  0.026  <0.0001  <0.0001  <0.0001  0.0002  0.0049  <0.0001  0.19  0.22 | 57.3 ± 5.3 Hz vs 42.3 ± 5.7 Hz  69.0 ± 4.4 Hz vs 55.9 ± 3.2 Hz  78.4 ± 3.2 Hz vs 56.7 ± 2.2 Hz  89.2 ± 2.3 Hz vs 61.5 ± 2.4 Hz  89.2 ± 2.3 Hz vs 57.3 ± 5.3 Hz  89.2 ± 2.3 Hz vs 69.0 ± 4.4 Hz  89.2 ± 2.3 Hz vs 78.4 ± 3.2 Hz  61.5 ± 2.4 Hz vs 42.3 ± 5.7 Hz  61.5 ± 2.4 Hz vs 55.9 ± 3.2 Hz  61.5 ± 2.4 Hz vs 56.8 ± 2.2 Hz | 20 cells vs 24 cells from 24 mice  17 cells vs 34 cells from 25 mice  43 cells vs 47 cells from 48 mice  70 cells vs 62 cells from 64 mice  70 cells vs 20 cells from 51 mice  70 cells vs 17 cells from 53 mice  70 cells vs 43 cells from 72 mice  62 cells vs 24 cells from 59 mice  62 cells vs 34 cells from 59 mice  62 cells vs 47 cells from 68 mice |
| Figure1 C_2_  Simple spike CV2 (*in vivo*) | Two-way ANOVA multiple comparisons | P12-P17 Z– vs P12-P17 Z+  P18-P29 Z– vs P18-P29 Z+  P30-P59 Z– vs P30-P59 Z+  >P60 Z– vs >P60 Z+ | 0.940  0.020  <0.0001  <0.001 | 0.51 ± 0.03 vs 0.51 ± 0.04  0.50 ± 0.02 vs 0.42 ± 0.02  0.44 ± 0.01 vs 0.34 ± 0.01  0.47 ± 0.01 vs 0.40 ± 0.01 | 20 cells vs 24 cells from 24 mice  17 cells vs 34 cells from 25 mice  43 cells vs 47 cells from 48 mice  70 cells vs 62 cells from 64 mice |
| Figure1 D_1_  Complex spike rate (*in vivo*) | Two-way ANOVA multiple comparisons | P12-P17 Z– vs P12-P17 Z+  P18-P29 Z– vs P18-P29 Z+  P30-P59 Z– vs P30-P59 Z+  >P60 Z– vs >P60 Z+  >P60 Z– vs P12-17 Z–  >P60 Z– vs P18-29 Z–  >P60 Z– vs P30-P59 Z–  >P60 Z+ vs P12-17 Z+  >P60 Z+ vs P18-29 Z+  >P60 Z+ vs P30-P59 + | 0.011  <0.001  0.019  <0.0001  <0.0001  0.005  0.86  0.16  0.010  0.06 | 0.79 ± 0.09 Hz vs 1.06 ± 0.11 Hz  1.50 ± 0.15 Hz vs 1.14 ± 0.08 Hz  1.25 ± 0.04 Hz vs 1.07 ± 0.04 Hz  1.24 ± 0.03 Hz vs 0.94 ± 0.04 Hz  1.24 ± 0.03 Hz vs 0.79 ± 0.09 Hz  1.24 ± 0.03 Hz vs 1.50 ± 0.16 Hz  1.24 ± 0.03 Hz vs 1.25 ± 0.04 Hz  0.94 ± 0.04 Hz vs 1.06 ± 0.11 Hz  0.94 ± 0.04 Hz vs 1.14 ± 0.08 Hz  0.94 ± 0.04 Hz vs 1.07 ± 0.04 Hz | 20 cells vs 24 cells from 24 mice  17 cells vs 34 cells from 25 mice  43 cells vs 47 cells from 48 mice  70 cells vs 62 cells from 64 mice  70 cells vs 20 cells from 51 mice  70 cells vs 17 cells from 53 mice  70 cells vs 43 cells from 72 mice  62 cells vs 24 cells from 59 mice  62 cells vs 34 cells from 59 mice  62 cells vs 47 cells from 68 mice |
| Figure1 D_2_  Climbing fiber pause (*in vivo*) | Kruskal-Wallis test  multiple comparisons | P12-P17 Z– vs P12-P17 Z+  P18-P29 Z– vs P18-P29 Z+  P30-P59 Z– vs P30-P59 Z+  >P60 Z– vs >P60 Z+ | 0.940  0.007  <0.0001  <0.0001 | 81.1 ± 12.7 msec vs 79.6 ± 13.8 msec  27.2 ± 6.1 msec vs 44.3 ± 5.6 msec  19.9 ± 1.6 msec vs 28.9 ± 1.9 msec  20.0 ± 0.8 msec vs 34.5 ± 2.4 msec | 20 cells vs 24 cells from 24 mice  17 cells vs 34 cells from 25 mice  43 cells vs 47 cells from 48 mice  70 cells vs 62 cells from 64 mice |

| **Related Figure** | **Test applied** | **Groups** | ***p*-value** | **Descriptive values** | **Number of cells / mice** |
| --- | --- | --- | --- | --- | --- |
| Figure2 B_1_  Simple spike rate (*in vitro*) | Two-way ANOVA multiple comparisons | P3-P5 LIII vs P3-P5 LX  P6-P9 LIII vs P6-P9 LX  P10-P11 LIII vs P10-P11 LX  P12-P17 LIII vs P12-P17 LX  P18-P29 LIII vs P18-P29 LX  P30-P59 LIII vs P30-P59 LX  >P60 LIII vs >P60 LX  P3-P5 LIII vs P10-P11 LIII  P3-P5 LX vs P10-P11 LX  >P60 Z– vs P3-5 Z–  >P60 Z– vs P6-9 Z–  >P60 Z– vs P10-11 Z–  >P60 Z– vs P12-17 Z–  >P60 Z– vs P18-29 Z–  >P60 Z– vs P30-P59 Z–  >P60 Z+ vs P3-5 Z+  >P60 Z+ vs P6-9 Z+  >P60 Z+ vs P10-11 Z+  >P60 Z+ vs P12-17 Z+  >P60 Z+ vs P18-29 Z+  >P60 Z+ vs P30-P59 + | 0.78  0.99  0.65  0.008  0.002  0.003  <0.0001  0.0002  0.0007  <0.0001  <0.0001  <0.0001  0.05  0.17  0.59  <0.0001  <0.0001  <0.0001  0.014  0.22  0.85 | 6.0 ± 0.5 Hz vs 4.1 ± 0.8 Hz  14.9 ± 1.3 Hz vs 14.8 ± 1.6 Hz  28.2 ± 1.5 Hz vs 25.9 ± 1.6 Hz  51.7 ± 3.4 Hz vs 34.8 ± 2.0 Hz  55.8 ± 5.6 Hz vs 42.4 ± 2.9 Hz  59.1 ± 4.8 Hz vs 45.6 ± 3.6 Hz  61.2 ± 2.5 Hz vs 46.3 ± 1.9 Hz  6.0 ± 0.5 Hz vs 28.2 ± 1.5 Hz  4.1 ± 0.8 Hz vs 25.9 ± 1.6 Hz  61.2 ± 2.5 Hz vs 6.0 ± 0.5 Hz  61.2 ± 2.5 Hz vs 14.9 ± 1.3 Hz  61.2 ± 2.5 Hz vs 28.2 ± 1.5 Hz  61.2 ± 2.5 Hz vs 51.7 ± 3.4 Hz  61.2 ± 2.5 Hz vs 55.8 ± 5.6 Hz  61.2 ± 2.5 Hz vs 59.1 ± 4.8 Hz  46.3 ± 1.9 Hz vs 4.1 ± 0.8 Hz  46.3 ± 1.9 Hz vs 14.8 ± 1.6 Hz  46.3 ± 1.9 Hz vs 25.9 ± 1.6 Hz  46.3 ± 1.9 Hz vs 34.8 ± 2.0 Hz  46.3 ± 1.9 Hz vs 42.4 ± 2.9 Hz  46.3 ± 1.9 Hz vs 45.6 ± 3.6 Hz | 23 cells vs 18 cells from 3 mice  30 cells vs 25 cells from 6 mice  37 cells vs 36 cells from 3 mice  23 cells vs 25 cells from 4 mice  43 cells vs 73 cells from 9 mice  44 cells vs 50 cells from 7 mice  102 cells vs 121 cells from 17 mice  23 cells vs 37 cells from 6 mice  18 cells vs 36 cells from 6 mice  102 cells vs 23 cells from 20 mice  102 cells vs 30 cells from 23 mice  102 cells vs 37 cells from 20 mice  102 cells vs 23 cells from 21 mice  102 cells vs 43 cells from 26 mice  102 cells vs 44 cells from 24 mice  121 cells vs 18 cells from 20 mice  121 cells vs 25 cells from 23 mice  121 cells vs 36 cells from 20 mice  121 cells vs 25 cells from 21 mice  121 cells vs 73 cells from 26 mice  121 cells vs 50 cells from 24 mice |
| Figure2 B_2_  Simple spike CV2 (*in vitro*) | Kruskal-Wallis test  multiple comparisons | P3-P5 LIII vs P3-P5 LX  P6-P9 LIII vs P6-P9 LX  P10-P11 LIII vs P10-P11 LX  P12-P17 LIII vs P12-P17 LX  P18-P29 LIII vs P18-P29 LX  P30-P59 LIII vs P30-P59 LX  >P60 LIII vs >P60 LX | 0.36  0.002  0.43  0.07  <0.0001  <0.0001  <0.0001 | 0.22 ± 0.04 vs 0.37 ± 0.07  0.11 ± 0.02 vs 0.22 ± 0.04  0.05 ± 0.00 vs 0.05 ± 0.00  0.05 ± 0.00 vs 0.09 ± 0.05  0.11 ± 0.01 vs 0.05 ± 0.00  0.09 ± 0.01 vs 0.06 ± 0.01  0.11 ± 0.01 vs 0.04 ± 0.00 | 23 cells vs 18 cells from 3 mice  30 cells vs 25 cells from 6 mice  37 cells vs 36 cells from 3 mice  23 cells vs 25 cells from 4 mice  43 cells vs 73 cells from 9 mice  44 cells vs 50 cells from 7 mice  102 cells vs 121 cells from 17 mice |

| **Related Figure** | **Test applied** | **Groups** | ***p*-value** | **Descriptive values** | **Number of cells / mice** |
| --- | --- | --- | --- | --- | --- |
| Figure3 B_1_  Dendritic intersections (sholl) | Two-way ANOVA multiple comparisons | P3-P5 LI-III vs P3-P5 LIX-X  P6-P11 LI-III vs P6-P11 LIX-X  P12-P17 LI-III vs P12-P17 LIX-X  P18-P29 LI-III vs P18-P29 LIX-X  P30-P59 LI-III vs P30-P59 LIX-X  >P60 LI-III vs >P60 LIX-X  >P60 LI-III vs P3-5 LI-III  >P60 LI-III vs P6-11 LI-III  >P60 LI-III vs P12-17 LI-III  >P60 LI-III vs P18-29 LI-III  >P60 LI-III vs P30-P59 LI-III  ->P60 LIX-X vs P3-5 LIX-X  >P60 LIX-X vs P6-11 LIX-X  >P60 LIX-X vs P12-17 LIX-X  >P60 LIX-X vs P18-29 LIX-X  >P60 LIX-X vs P30-P59 LIX-X | 0.79  0.52  0.38  <0.0001  <0.0001  <0.0001  <0.0001  <0.0001  <0.0001  0.002  0.68  <0.0001  <0.0001  0.0003  0.0007  0.002 | 26.2 ± 4.0 vs 42.77 ± 9.1  86.8 ± 9.2 vs 64.1 ± 7.3  336.1 ± 31.7 vs 291.2 ± 17.1  530.9 ± 41.6 vs 304.5 ± 31.3  651.3 ± 25.4 vs 351.6 ± 28.1  663.1 ± 23.8 vs 445.7 ± 22.2  663.1 ± 23.8 vs 26.2 ± 4.0  663.1 ± 23.8 vs 86.8 ± 9.2  663.1 ± 23.8 vs 336.1 ± 31.7  663.1 ± 23.8 vs 531.0 ± 41.6  663.1 ± 23.8 vs 651.3 ± 25.4  445.7 ± 22.2 vs 42.7 ± 9.1  445.7 ± 22.2 vs 64.1 ± 7.3  445.7 ± 22.2 vs 291.2 ± 17.1  445.7 ± 22.2 vs 304.5 ± 31.3  445.7 ± 22.2 vs 351.6 ± 28.1 | 9 cells vs 12 cells from 3 mice  28 cells vs 38 cells from 9 mice  18 cells vs 14 cells from 4 mice  14 cells vs 15 cells from 9 mice  38 cells vs 35 cells from 7 mice  61 cells vs 59 cells from 17 mice  61 cells vs 9 cells from 33 mice  61 cells vs 28 cells from 38 mice  61 cells vs 18 cells from 34 mice  61 cells vs 14 cells from 33 mice  61 cells vs 38 cells from 43 mice  59 cells vs 12 cells from 31 mice  59 cells vs 38 cells from 36 mice  59 cells vs 14 cells from 29 mice  59 cells vs 15 cells from 32 mice  59 cells vs 35 cells from 40 mice |
| Figure3 B_2_  Dendritic length from soma | Two-way ANOVA multiple comparisons | P3-P5 LI-III vs P3-P5 LIX-X  P6-P11 LI-III vs P6-P11 LIX-X  P12-P17 LI-III vs P12-P17 LIX-X  P18-P29 LI-III vs P18-P29 LIX-X  P30-P59 LI-III vs P30-P59 LIX-X  >P60 LI-III vs >P60 LIX-X | 0.45  0.12  0.74  0.012  <0.0001  <0.0001 | 32.8 ± 2.9 µm vs 44.6 ± 5.5 µm  73.0 ± 4.8 µm vs 89.3 ± 7.0 µm  138.3 ± 5.1 µm vs 142.5 ± 3.9 µm  193.9 ± 12.8 µm vs 160.7 ± 11.0 µm  215.1 ± 6.7 µm vs 160.3 ± 5.7 µm  206.6 ± 5.9 µm vs 169.9 ± 4.0 µm | 9 cells vs 12 cells from 3 mice  28 cells vs 38 cells from 9 mice  18 cells vs 14 cells from 4 mice  14 cells vs 15 cells from 9 mice  38 cells vs 35 cells from 7 mice  61 cells vs 59 cells from 17 mice |
| Figure3 B_3_  Dendritic area size | Two-way ANOVA multiple comparisons | P3-P5 LI-III vs P3-P5 LIX-X  P6-P11 LI-III vs P6-P11 LIX-X  P12-P17 LI-III vs P12-P17 LIX-X  P18-P29 LI-III vs P18-P29 LIX-X  P30-P59 LI-III vs P30-P59 LIX-X  >P60 LI-III vs >P60 LIX-X | 0.92  0.62  0.46  <0.0001  <0.0001  <0.0001 | 536 ± 45 µm^2^ vs 648.8 ± 102 µm^2^  1807 ± 187 µm^2^ vs 1489 ± 139 µm^2^  5419 ± 399 µm^2^ vs 6109 ± 462 µm^2^  10541 ± 761 µm^2^ vs 6200 ± 603 µm^2^  13011 ± 583 µm^2^ vs 7763 ± 499 µm^2^  10890 ± 412 µm^2^ vs 8520 ± 377 µm^2^ | 9 cells vs 12 cells from 3 mice  28 cells vs 38 cells from 9 mice  18 cells vs 14 cells from 4 mice  14 cells vs 15 cells from 9 mice  38 cells vs 35 cells from 7 mice  61 cells vs 59 cells from 17 mice |
| Figure4 B_1_  Molecular layer thickness | Two-way ANOVA multiple comparisons | P7 LI-III vs P7 LIX-X  P14 LI-III vs P14 LIX-X  P21 LI-III vs P21 LIX-X  P35 LI-III vs P35 LIX-X  P60 LI-III vs P60 LIX-X | 0.16  0.78  0.002  0.05  <0.001 | 21.8 ± 1.8 µm vs 28.6 ± 0.2 µm  101.5 ± 3.3 µm vs 102.9 ± 3.6 µm  118.9 ± 2.9 µm vs 101.8 ± 0.3 µm  126.3 ± 6.8 µm vs 116.7 ± 1.8 µm  137.5 ± 1.3 µm vs 116.9 ± 4.9 µm | 3 mice  3 mice  3 mice  3 mice  3 mice |

| **Related Figure** | **Test applied** | **Groups** | ***p*-value** | **Descriptive values** | | **Number of cells / mice** | |
| --- | --- | --- | --- | --- | --- | --- | --- |
| Figure4 B_2_  Molecular layer VGluT2 puncta | Two-way ANOVA multiple comparisons | P7 LI-III vs P7 LIX-X  P14 LI-III vs P14 LIX-X  P21 LI-III vs P21 LIX-X  P35 LI-III vs P35 LIX-X  P60 LI-III vs P60 LIX-X | <0.0001  0.78  0.79  0.88  0.76 | 0.62 ± 0.31 % vs 3.87 ± 0.19 %  1.67 ± 0.17 % vs 1.51 ± 0.23 %  1.26 ± 0.31 % vs 1.41 ± 0.11 %  3.50 ± 0.98 % vs 3.58 ± 0.35 %  2.74 ± 0.23 % vs 2.91 ± 0.13 % | | 3 mice  3 mice  3 mice  3 mice  3 mice | |
| Figure4 B_3_  Climbing fiber coverage of molecular layer | Two-way ANOVA multiple comparisons | P7 LI-III vs P7 LIX-X  P14 LI-III vs P14 LIX-X  P21 LI-III vs P21 LIX-X  P35 LI-III vs P35 LIX-X  P60 LI-III vs P60 LIX-X  P60 LI-III vs P7 LI-III  P60 LI-III vs P14 LI-III  P60 LI-III vs P21 LI-III  P60 LI-III vs P35 LI-III  P60 LIX-X vs P7 LIX-X  P60 LIX-X vs P14 LIX-X  P60 LIX-X vs P21 LIX-X  P60 LIX-X vs P35 LIX-X | <0.0001  0.24  0.41  0.36  0.66  <0.0001  <0.0001  0.002  0.54  0.89  0.002  0.001  0.90 | 11.1 ± 1.1 % vs 80.5 ± 1.0 %  62.4 ± 1.1 % vs 66.9 ± 7.8 %  69.1 ± 0.3 % vs 66.0 ± 1.7 %  85.1 ± 1.0 % vs 81.5 ± 1.1 %  82.7 ± 0.6 % vs 81.0 ± 0.4 %  82.7 ± 0.6 % vs 11.1 ± 1.1 %  82.7 ± 0.6 % vs 62.4 ± 1.1 %  82.7 ± 0.6 % vs 69.1 ± 0.3 %  82.7 ± 0.6 % vs 85.1 ± 1.0 %  81.0 ± 0.4 % vs 80.5 ± 1.0 %  81.0 ± 0.4 % vs 66.9 ± 7.8 %  81.0 ± 0.4 % vs 66.0 ± 1.7 %  81.0 ± 0.4 % vs 81.5 ± 1.1 % | | 3 mice  3 mice  3 mice  3 mice  3 mice  3 mice vs 3 mice  3 mice vs 3 mice  3 mice vs 3 mice  3 mice vs 3 mice  3 mice vs 3 mice  3 mice vs 3 mice  3 mice vs 3 mice  3 mice vs 3 mice | |
| Figure5 D_1_  Axonal intersections (sholl) P10 | Two-way ANOVA multiple comparisons | P10 Z– vs P10 Z+ | 0.045 | 233.0 ± 42.4 vs 273.25 ± 68.5 | | 7 axons vs 8 axons from 8 mice | |
| Figure5 D_2_  Axonal intersections (sholl) P14 | Two-way ANOVA multiple comparisons | P14 Z– vs P14 Z+ | 0.001 | 246.3 ± 22.8 vs 293.1 ± 24.6 | | 11 axons vs 20 axons from 10 mice | |
| Figure5 D_3_  Axonal intersections (sholl) P21 | Two-way ANOVA multiple comparisons | P21 Z– vs P21 Z+ | 0.038 | 274.2 ± 73.5 vs 306.6 ± 42.5 | | 8 axons vs 18 axons from 7 mice | |
| Figure5 E_1_  Axonal intersections (sholl) | One-way ANOVA  multiple comparisons | P7, P10, P14, P21 | 0.013 | 84.2 ± 18.2, 254.4 ± 40.5,  277.4 ± 17.5, 296.4 ± 37.9 | | 6, 15, 31, 26 axons from 29 mice | |
| **Related Figure** | **Test applied** | **Groups** | ***p*-value** | **Descriptive values** | | **Number of cells / mice** | |
| Figure5 E_2_  Axonal length from first intersection | One-way ANOVA  multiple comparisons | P7, P10, P14, P21 | 0.060 | 124.2 ± 17.8 µm, 212.6 ± 24.3 µm,  216.4 ± 14.3 µm, 225.2 ± 16.1 µm | | 6, 15, 31, 26 axons from 29 mice | |
| Figure5 E_3_  Axonal area size | One-way ANOVA  multiple comparisons | P7, P10, P14, P21 | <0.0001 | 1.52 ± 0.20 µm^2^·10^3^, 1.46 ± 0.24 µm^2^·10^3^, 2.26 ± 0.19 µm^2^·10^3^, 3.07 ± 0.29 µm^2^·10^3^ | | 6, 15, 31, 26 axons from 29 mice | |
| Figure5 F_1_  Axonal intersections (sholl) | Two-way ANOVA multiple comparisons | Interaction effect Z– vs Z+  P10 Z– vs P10 Z+  P14 Z– vs P14 Z+  P21 Z– vs P21 Z+  P21 Z– vs P10 Z–  P21 Z– vs P14 Z–  P21 Z+ vs P10 Z+  P21 Z+ vs P14 Z+ | 0.25  0.97  0.97  0.99  0.96  >0.99  >0.99  >0.99 | 233.0 ± 42.4 vs 273.25 ± 68.5  246.3 ± 22.8 vs 293.1 ± 24.6  274.2 ± 73.5 vs 306.6 ± 42.5  274.2 ± 73.5 vs 233.0 ± 42.4  274.2 ± 73.5 vs 246.3 ± 22.8  306.6 ± 42.5 vs 273.25 ± 68.5  306.6 ± 42.5 vs 293.1 ± 24.6 | | 7 axons vs 8 axons from 8 mice  11 axons vs 20 axons from 10 mice  8 axons vs 18 axons from 7 mice  8 axons vs 7 axons from 41 mice  8 axons vs 11 axons from 57 mice  18 axons vs 8 axons from 41 mice  18 axons vs 20 axons from 57 mice | |
| Figure5 F_2_  Axonal length from first intersection | Two-way ANOVA multiple comparisons | Interaction effect Z– vs Z+  P10 Z– vs P10 Z+  P14 Z– vs P14 Z+  P21 Z– vs P21 Z+ | 0.019  0.83  0.54  0.74 | 200.7 ± 35.4 µm^2^ vs 223.1 ± 35.3 µm^2^  197.3 ± 25.7 µm^2^ vs 227.7 ± 18.1 µm^2^  188.1 ± 28.7 µm^2^ vs 241.1 ± 17.7 µm^2^ | | 7 axons vs 8 axons from 8 mice  11 axons vs 20 axons from 10 mice  8 axons vs 18 axons from 7 mice | |
| Figure5 F_3_  Axonal area size | Two-way ANOVA multiple comparisons | Interaction effect Z– vs Z+  P10 Z– vs P10 Z+  P14 Z– vs P14 Z+  P21 Z– vs P21 Z+ | 0.025  0.82  >0.99  0.14 | 1.17 ± 0.18 µm^2^·10^3^ vs 1.73 ± 0.42 µm^2^·10^3^  2.14 ± 0.26 µm^2^·10^3^ vs 2.37 ± 0.28 µm^2^·10^3^  2.36 ± 0.46 µm^2^·10^3^ vs 3.39 ± 0.33 µm^2^·10^3^ | | 7 axons vs 8 axons from 8 mice  11 axons vs 20 axons from 10 mice  8 axons vs 18 axons from 7 mice | |
| Figure6 A_2_  VOR phase reversal | Mixed-effects repeated measures | Young vs Adult day1  Young vs Adult day2  Young vs Adult day3  Young vs Adult day4  Young vs Adult day5 | <0.0001  <0.0001  <0.0001  <0.0001  <0.0001 | 25.7 ± 0.9 ° vs 18.7 ± 1.1 °  100.4 ± 15.7 ° vs 29.6 ± 2.9 °  162.9 ± 9.9 ° vs 56.4 ± 7.0 °  174.8 ± 1.5 ° vs 102.3 ± 8.7 °  175.9 ± 0.7 ° vs 126.4 ± 7.0 ° | | 16 mice vs 11 mice | |
| Figure6 B_2_  Eyeblink conditioned response | Mixed-effects repeated measures | Young vs Adult | 0.027 | 20.4 ± 4.4 % vs 30.9 ± 6.0 % | | 18 mice vs 13 mice | |
| **Related Figure** | **Test applied** | **Groups** | ***p*-value** | **Descriptive values** | **Number of cells / mice** | |  |
| Figure 7  Simple spike rate *(in vitro)* | Two-way ANOVA multiple comparisons | >P60 Z– vs P3-5 Z–  >P60 Z– vs P6-9 Z–  >P60 Z– vs P10-11 Z–  >P60 Z– vs P12-17 Z–  >P60 Z– vs P18-29 Z–  >P60 Z– vs P30-P59 Z–  >P60 Z+ vs P3-5 Z+  >P60 Z+ vs P6-9 Z+  >P60 Z+ vs P10-11 Z+  >P60 Z+ vs P12-17 Z+  >P60 Z+ vs P18-29 Z+  >P60 Z+ vs P30-P59 + | <0.0001  <0.0001  <0.0001  0.05  0.17  0.59  <0.0001  <0.0001  <0.0001  0.014  0.22  0.85 | 61.2 ± 2.5 Hz vs 6.0 ± 0.5 Hz  61.2 ± 2.5 Hz vs 14.9 ± 1.3 Hz  61.2 ± 2.5 Hz vs 28.2 ± 1.5 Hz  61.2 ± 2.5 Hz vs 51.7 ± 3.4 Hz  61.2 ± 2.5 Hz vs 55.8 ± 5.6 Hz  61.2 ± 2.5 Hz vs 59.1 ± 4.8 Hz  46.3 ± 1.9 Hz vs 4.1 ± 0.8 Hz  46.3 ± 1.9 Hz vs 14.8 ± 1.6 Hz  46.3 ± 1.9 Hz vs 25.9 ± 1.6 Hz  46.3 ± 1.9 Hz vs 34.8 ± 2.0 Hz  46.3 ± 1.9 Hz vs 42.4 ± 2.9 Hz  46.3 ± 1.9 Hz vs 45.6 ± 3.6 Hz | 102 cells vs 23 cells from 20 mice  102 cells vs 30 cells from 23 mice  102 cells vs 37 cells from 20 mice  102 cells vs 23 cells from 21 mice  102 cells vs 43 cells from 26 mice  102 cells vs 44 cells from 24 mice  121 cells vs 18 cells from 20 mice  121 cells vs 25 cells from 23 mice  121 cells vs 36 cells from 20 mice  121 cells vs 25 cells from 21 mice  121 cells vs 73 cells from 26 mice  121 cells vs 50 cells from 24 mice | |  |
| Figure 7  Simple spike rate *(in vivo)* | Two-way ANOVA multiple comparisons | >P60 Z– vs P12-17 Z–  >P60 Z– vs P18-29 Z–  >P60 Z– vs P30-P59 Z–  >P60 Z+ vs P12-17 Z+  >P60 Z+ vs P18-29 Z+  >P60 Z+ vs P30-P59 + | <0.0001  0.0002  0.0049  <0.0001  0.19  0.22 | 89.2 ± 2.3 Hz vs 57.3 ± 5.3 Hz  89.2 ± 2.3 Hz vs 69.0 ± 4.4 Hz  89.2 ± 2.3 Hz vs 78.4 ± 3.2 Hz  61.5 ± 2.4 Hz vs 42.3 ± 5.7 Hz  61.5 ± 2.4 Hz vs 55.9 ± 3.2 Hz  61.5 ± 2.4 Hz vs 56.8 ± 2.2 Hz | 70 cells vs 20 cells from 51 mice  70 cells vs 17 cells from 53 mice  70 cells vs 43 cells from 72 mice  62 cells vs 24 cells from 59 mice  62 cells vs 34 cells from 59 mice  62 cells vs 47 cells from 68 mice | |  |
| Figure 7  Complex spike rate *(in vivo)* | Two-way ANOVA multiple comparisons | >P60 Z– vs P12-17 Z–  >P60 Z– vs P18-29 Z–  >P60 Z– vs P30-P59 Z–  >P60 Z+ vs P12-17 Z+  >P60 Z+ vs P18-29 Z+  >P60 Z+ vs P30-P59 + | <0.0001  0.005  0.86  0.16  0.010  0.06 | 1.24 ± 0.03 Hz vs 0.79 ± 0.09 Hz  1.24 ± 0.03 Hz vs 1.50 ± 0.16 Hz  1.24 ± 0.03 Hz vs 1.25 ± 0.04 Hz  0.94 ± 0.04 Hz vs 1.06 ± 0.11 Hz  0.94 ± 0.04 Hz vs 1.14 ± 0.08 Hz  0.94 ± 0.04 Hz vs 1.07 ± 0.04 Hz | 70 cells vs 20 cells from 51 mice  70 cells vs 17 cells from 53 mice  70 cells vs 43 cells from 72 mice  62 cells vs 24 cells from 59 mice  62 cells vs 34 cells from 59 mice  62 cells vs 47 cells from 68 mice | |  |
| Figure 7  Dendritic intersections (sholl) | Two-way ANOVA multiple comparisons | >P60 LI-III vs P3-5 LI-III  >P60 LI-III vs P6-11 LI-III  >P60 LI-III vs P12-17 LI-III  >P60 LI-III vs P18-29 LI-III  >P60 LI-III vs P30-P59 LI-III  ->P60 LIX-X vs P3-5 LIX-X  >P60 LIX-X vs P6-11 LIX-X  >P60 LIX-X vs P12-17 LIX-X  >P60 LIX-X vs P18-29 LIX-X  >P60 LIX-X vs P30-P59 LIX-X | <0.0001  <0.0001  <0.0001  0.002  0.68  <0.0001  <0.0001  0.0003  0.0007  0.002 | 663.1 ± 23.8 vs 26.2 ± 4.0  663.1 ± 23.8 vs 86.8 ± 9.2  663.1 ± 23.8 vs 336.1 ± 31.7  663.1 ± 23.8 vs 531.0 ± 41.6  663.1 ± 23.8 vs 651.3 ± 25.4  445.7 ± 22.2 vs 42.7 ± 9.1  445.7 ± 22.2 vs 64.1 ± 7.3  445.7 ± 22.2 vs 291.2 ± 17.1  445.7 ± 22.2 vs 304.5 ± 31.3  445.7 ± 22.2 vs 351.6 ± 28.1 | 61 cells vs 9 cells from 33 mice  61 cells vs 28 cells from 38 mice  61 cells vs 18 cells from 34 mice  61 cells vs 14 cells from 33 mice  61 cells vs 38 cells from 43 mice  59 cells vs 12 cells from 31 mice  59 cells vs 38 cells from 36 mice  59 cells vs 14 cells from 29 mice  59 cells vs 15 cells from 32 mice  59 cells vs 35 cells from 40 mice | |  |

| **Related Figure** | **Test applied** | **Groups** | ***p*-value** | **Descriptive values** | **Number of cells / mice** |
| --- | --- | --- | --- | --- | --- |
| Figure 7  Axonal intersections (sholl) | Two-way ANOVA multiple comparisons | P21 Z– vs P10 Z–  P21 Z– vs P14 Z–  P21 Z+ vs P10 Z+  P21 Z+ vs P14 Z+ | 0.96  >0.99  >0.99  >0.99 | 274.2 ± 73.5 vs 233.0 ± 42.4  274.2 ± 73.5 vs 246.3 ± 22.8  306.6 ± 42.5 vs 273.25 ± 68.5  306.6 ± 42.5 vs 293.1 ± 24.6 | 8 axons vs 7 axons from 41 mice  8 axons vs 11 axons from 57 mice  18 axons vs 8 axons from 41 mice  18 axons vs 20 axons from 57 mice |
| Figure 7  Climbing fiber coverage of molecular layer | Two-way ANOVA multiple comparisons | P60 LI-III vs P7 LI-III  P60 LI-III vs P14 LI-III  P60 LI-III vs P21 LI-III  P60 LI-III vs P35 LI-III  P60 LIX-X vs P7 LIX-X  P60 LIX-X vs P14 LIX-X  P60 LIX-X vs P21 LIX-X  P60 LIX-X vs P35 LIX-X | <0.0001  <0.0001  0.002  0.54  0.89  0.002  0.001  0.90 | 82.7 ± 0.6 % vs 11.1 ± 1.1 %  82.7 ± 0.6 % vs 62.4 ± 1.1 %  82.7 ± 0.6 % vs 69.1 ± 0.3 %  82.7 ± 0.6 % vs 85.1 ± 1.0 %  81.0 ± 0.4 % vs 80.5 ± 1.0 %  81.0 ± 0.4 % vs 66.9 ± 7.8 %  81.0 ± 0.4 % vs 66.0 ± 1.7 %  81.0 ± 0.4 % vs 81.5 ± 1.1 % | 3 mice vs 3 mice  3 mice vs 3 mice  3 mice vs 3 mice  3 mice vs 3 mice  3 mice vs 3 mice  3 mice vs 3 mice  3 mice vs 3 mice  3 mice vs 3 mice |
| Supplement Figure1.1 B_1_  Simple spike CV | Kruskal-Wallis test  multiple comparisons | P12-P17 Z– vs P12-P17 Z+  P18-P29 Z– vs P18-P29 Z+  P30-P59 Z– vs P30-P59 Z+  >P60 Z– vs >P60 Z+  P12-P17 Z– vs >P60 Z–  P12-P17 Z+ vs >P60 Z+ | >0.99  0.79  0.27  <0.0001  <0.0001  <0.0001 | 2.12 ± 0.31 vs 2.10 ± 0.35  0.99 ± 0.18 vs 0.99 ± 0.18  0.60 ± 0.12 vs 0.51 ± 0.10  0.68 ± 0.03 vs 0.53 ± 0.05  2.12 ± 0.31 vs 0.68 ± 0.03  2.10 ± 0.35 vs 0.53 ± 0.05 | 20 cells vs 24 cells from 24 mice  17 cells vs 34 cells from 25 mice  43 cells vs 47 cells from 48 mice  70 cells vs 62 cells from 64 mice  20 cells vs 70 cells from 51 mice  24 cells vs 62 cells from 59 mice |
| Supplement Figure1.1 B_2_  Simple spike regularity index | Kruskal-Wallis test  multiple comparisons | P12-P17 Z– vs P12-P17 Z+  P18-P29 Z– vs P18-P29 Z+  P30-P59 Z– vs P30-P59 Z+  >P60 Z– vs >P60 Z+  P12-P17 Z– vs >P60 Z–  P12-P17 Z+ vs >P60 Z+ | 0.29  0.17  0.011  0.24  <0.0001  <0.0001 | 0.19 ± 0.03 vs 0.23 ± 0.03  0.10 ± 0.02 vs 0.11 ± 0.02  0.06 ± 0.01 vs 0.11 ± 0.02  0.01 ± 0.00 vs 0.01 ± 0.00  0.19 ± 0.03 vs 0.01 ± 0.00  0.23 ± 0.03 vs 0.01 ± 0.00 | 20 cells vs 24 cells from 24 mice  17 cells vs 34 cells from 25 mice  43 cells vs 47 cells from 48 mice  70 cells vs 62 cells from 64 mice  20 cells vs 70 cells from 51 mice  24 cells vs 62 cells from 59 mice |
| Supplement Figure1.1 C_1_  Complex spike CV2 | Two-way ANOVA multiple comparisons | P12-P17 Z– vs P12-P17 Z+  P18-P29 Z– vs P18-P29 Z+  P30-P59 Z– vs P30-P59 Z+  >P60 Z– vs >P60 Z+ | 0.015  0.86  0.22  0.034 | 0.91 ± 0.03 vs 0.85 ± 0.02  0.82 ± 0.02 vs 0.82 ± 0.02  0.85 ± 0.01 vs 0.82 ± 0.01  0.84 ± 0.01 vs 0.81 ± 0.01 | 20 cells vs 24 cells from 24 mice  17 cells vs 34 cells from 25 mice  43 cells vs 47 cells from 48 mice  70 cells vs 62 cells from 64 mice |

| **Related Figure** | **Test applied** | **Groups** | ***p*-value** | **Descriptive values** | **Number of cells / mice** |
| --- | --- | --- | --- | --- | --- |
| Supplement Figure1.3 A_1_  Simple spike rate P>60 | One-way ANOVA  multiple comparisons | P>60 LI-II vs P>60 LIII  P>60 LI-II vs P>60 LIV-V  P>60 LI-II vs P>60 LVI-VII  P>60 LI-II vs P>60 LIX  P>60 LI-II vs P>60 LX  P>60 LIII vs P>60 LIV-V  P>60 LIII vs P>60 LVI-VII  P>60 LIII vs P>60 LIX  P>60 LIII vs P>60 LX  P>60 LIV-V vs P>60 LVI-VII  P>60 LIV-V vs P>60 LIX  P>60 LIV-V vs P>60 LX  P>60 LVI-VII vs P>60 LIX  P>60 LVI-VII vs P>60 LX  P>60 LIX vs P>60 LX | 0.44  0.009  0.53  0.002  <0.0001  0.037  0.97  0.013  <0.0001  0.09  0.92  0.08  0.06  0.0005  0.018 | 91.9 ± 5.3 Hz vs 87.0 ± 3.9 Hz  91.9 ± 5.3 Hz vs 71.3 ± 7.8 Hz  91.9 ± 5.3 Hz vs 86.7 ± 8.4 Hz  91.9 ± 5.3 Hz vs 72.0 ± 3.1 Hz  91.9 ± 5.3 Hz vs 58.7 ± 3.6 Hz  87.0 ± 3.9 Hz vs 71.3 ± 7.8 Hz  87.0 ± 3.9 Hz vs 86.7 ± 8.4 Hz  87.0 ± 3.9 Hz vs 72.0 ± 3.1 Hz  87.0 ± 3.9 Hz vs 58.7 ± 3.6 Hz  71.3 ± 7.8 Hz vs 86.7 ± 8.4 Hz  71.3 ± 7.8 Hz vs 72.0 ± 3.1 Hz  71.3 ± 7.8 Hz vs 58.7 ± 3.6 Hz  86.7 ± 8.4 Hz vs 72.0 ± 3.1 Hz  86.7 ± 8.4 Hz vs 58.7 ± 3.6 Hz  72.0 ± 3.1 Hz vs 58.7 ± 3.6 Hz | 19 cells vs 22 cells from 22 mice  19 cells vs 11 cells from 17 mice  19 cells vs 9 cells from 17 mice  19 cells vs 25 cells from 23 mice  19 cells vs 28 cells from 33 mice  22 cells vs 11 cells from 21 mice  22 cells vs 9 cells from 21 mice  22 cells vs 25 cells from 27 mice  22 cells vs 28 cells from 37 mice  11 cells vs 9 cells from 16 mice  11 cells vs 25 cells from 22 mice  11 cells vs 28 cells from 32 mice  9 cells vs 25 cells from 22 mice  9 cells vs 28 cells from 32 mice  25 cells vs 28 cells from 38 mice |
| Supplement Figure1.3 A_2_  Simple spike rate P30-59 | One-way ANOVA  multiple comparisons | P30-59 LI-II vs P30-59 LIII  P30-59 LI-II vs P30-59 LIV-V  P30-59 LI-II vs P30-59 LVI-VII  P30-59 LI-II vs P30-59 LVIII  P30-59 LI-II vs P30-59 LIX  P30-59 LI-II vs P30-59 LX  P30-59 LIII vs P30-59 LIV-V  P30-59 LIII vs P30-59 LVI-VII  P30-59 LIII vs P30-59 LVIII  P30-59 LIII vs P30-59 LIX  P30-59 LIII vs P30-59 LX  P30-59 LIV-V vs P30-59 LVI-VII  P30-59 LIV-V vs P30-59 LVIII  P30-59 LIV-V vs P30-59 LIX  P30-59 LIV-V vs P30-59 LX  P30-59 LVI-VII vs P30-59 LVIII  P30-59 LVI-VII vs P30-59 LIX  P30-59 LVI-VII vs P30-59 LX  P30-59 LVIII vs P30-59 LIX  P30-59 LVIII vs P30-59 LX  P30-59 LIX vs P30-59 LX | 0.83  0.38  0.35  0.52  0.024  0.007  0.72  0.66  0.51  0.09  0.05  0.87  0.14  0.0009  0.0001  0.14  0.002  0.0005  0.15  0.07  0.68 | 73.4 ± 5.7 Hz vs 75.7 ± 4.7 Hz  73.4 ± 5.7 Hz vs 79.5 ± 4.1 Hz  73.4 ± 5.7 Hz vs 80.7 ± 6.7 Hz  73.4 ± 5.7 Hz vs 68.3 ± 2.9 Hz  73.4 ± 5.7 Hz vs 57.9 ± 5.0 Hz  73.4 ± 5.7 Hz vs 55.6 ± 3.7 Hz  75.7 ± 4.7 Hz vs 79.5 ± 4.1 Hz  75.7 ± 4.7 Hz vs 80.7 ± 6.7 Hz  75.7 ± 4.7 Hz vs 68.3 ± 2.9 Hz  75.7 ± 4.7 Hz vs 57.9 ± 5.0 Hz  75.7 ± 4.7 Hz vs 55.6 ± 3.7 Hz  79.5 ± 4.1 Hz vs 80.7 ± 6.7 Hz  79.5 ± 4.1 Hz vs 68.3 ± 2.9 Hz  79.5 ± 4.1 Hz vs 57.9 ± 5.0 Hz  79.5 ± 4.1 Hz vs 55.6 ± 3.7 Hz  80.7 ± 6.7 Hz vs 68.3 ± 2.9 Hz  80.7 ± 6.7 Hz vs 57.9 ± 5.0 Hz  80.7 ± 6.7 Hz vs 55.6 ± 3.7 Hz  68.3 ± 2.9 Hz vs 57.9 ± 5.0 Hz  68.3 ± 2.9 Hz vs 55.6 ± 3.7 Hz  57.9 ± 5.0 Hz vs 55.6 ± 3.7 Hz | 10 cells vs 3 cells from 12 mice  10 cells vs 13 cells from 20 mice  10 cells vs 8 cells from 16 mice  10 cells vs 8 cells from 16 mice  10 cells vs 15 cells from 21 mice  10 cells vs 20 cells from 24 mice  3 cells vs 13 cells from 14 mice  3 cells vs 8 cells from 10 mice  3 cells vs 8 cells from 10 mice  3 cells vs 15 cells from 15 mice  3 cells vs 20 cells from 18 mice  13 cells vs 8 cells from 18 mice  13 cells vs 8 cells from 18 mice  13 cells vs 15 cells from 23 mice  13 cells vs 20 cells from 26 mice  8 cells vs 8 cells from 14 mice  8 cells vs 15 cells from 19 mice  8 cells vs 20 cells from 22 mice  8 cells vs 15 cells from 19 mice  8 cells vs 20 cells from 22 mice  15 cells vs 20 cells from 27 mice |

| **Related Figure** | **Test applied** | | | **Groups** | | ***p*-value** | | **Descriptive values** | | **Number of cells / mice** | |  |
| --- | --- | --- | --- | --- | --- | --- | --- | --- | --- | --- | --- | --- |
| Supplement Figure 1.3 A_3_  Simple spike rate P18-29 | | | One-way ANOVA  multiple comparisons | | P18-29 LI-II vs P18-29 LIII  P18-29 LI-II vs P18-29 LIV-V  P18-29 LI-II vs P18-29 LIX  P18-29 LI-II vs P18-29 LX  P18-29 LIII vs P18-29 LIV-V  P18-29 LIII vs P18-29 LIX  P18-29 LIII vs P18-29 LX  P18-29 LIV-V vs P18-29 LIX  P18-29 LIV-V vs P18-29 LX  P18-29 LIX vs P18-29 LX | | 0.76  0.07  0.61  0.84  0.12  0.90  0.57  0.06  0.015  0.30 | | 55.4 ± 12.1 Hz vs 59.7 ± 17.0 Hz  55.4 ± 12.1 Hz vs 80.7 ± 13.5 Hz  55.4 ± 12.1 Hz vs 61.1 ± 3.1 Hz  55.4 ± 12.1 Hz vs 53.0 ± 5.2 Hz  59.7 ± 17.0 Hz vs 80.7 ± 13.5 Hz  59.7 ± 17.0 Hz vs 61.1 ± 3.1 Hz  59.7 ± 17.0 Hz vs 53.0 ± 5.2 Hz  80.7 ± 13.5 Hz vs 61.1 ± 3.1 Hz  80.7 ± 13.5 Hz vs 53.0 ± 5.2 Hz  61.1 ± 3.1 Hz vs 53.0 ± 5.2 Hz | | 3 cells vs 3 cells from 6 mice  3 cells vs 4 cells from 6 mice  3 cells vs 13 cells from 12 mice  3 cells vs 8 cells from 11 mice  3 cells vs 4 cells from 6 mice  3 cells vs 13 cells from 12 mice  3 cells vs 8 cells from 11 mice  4 cells vs 13 cells from 12 mice  4 cells vs 8 cells from 11 mice  13 cells vs 8 cells from 17 mice |  |
| Supplement Figure1.3 A_4_  Simple spike rate P12-17 | | One-way ANOVA  multiple comparisons | | | P12-17 LIV-V vs P12-17 LVI-VII  P12-17 LIV-V vs P12-17 LX  P12-17 LVI-VII vs P12-17 LX | | 0.76  0.02  0.03 | | 62.2 ± 12.0 Hz vs 67.9 ± 21.3 Hz  62.2 ± 12.0 Hz vs 24.9 ± 3.8 Hz  67.9 ± 21.3 Hz vs 24.9 ± 3.8 Hz | | 7 cells vs 3 cells from 9 mice  7 cells vs 7 cells from 10 mice  3 cells vs 7 cells from 7 mice | |
| Supplement Figure1.3 B_1_  Simple spike rate ZebrinII- anterior and hemisphere | | One-way ANOVA  multiple comparisons | | | >P60 Ant. Z– vs P30-59 Ant. Z–  >P60 Ant. Z– vs P18-29 Ant. Z–  >P60 Ant. Z– vs P12-17 Ant. Z–  >P60 Hem. Z– vs P30-59 Hem. Z–  >P60 Hem. Z– vs P18-29 Hem. Z–  >P60 Hem. Z– vs P12-17 Hem. Z– | | 0.03  0.13  0.001  0.74  0.01  <0.0001 | | 87.7 ± 2.9 Hz vs 76.7 ± 3.0 Hz  87.7 ± 2.9 Hz vs 76.0 ± 6.9 Hz  87.7 ± 2.9 Hz vs 61.2 ± 10.5 Hz  91.6 ± 7.7 Hz vs 95.8 ± 19.7 Hz  91.6 ± 7.7 Hz vs 63.5 ± 6.8 Hz  91.6 ± 7.7 Hz vs 44.5 ± 4.6 Hz | | 50 cells vs 26 cells from 53 mice  50 cells vs 8 cells from 37 mice  50 cells vs 8 cells from 37 mice  8 cells vs 4 cells from 11 mice  8 cells vs 6 cells from 13 mice  8 cells vs 7 cells from 13 mice | |
| Supplement Figure1.3 B_2_  Simple spike rate ZebrinII+ Nodulus and Flocculus | | One-way ANOVA  multiple comparisons | | | >P60 Nod. Z+ vs P30-59 Nod. Z+  >P60 Nod. Z+ vs P18-29 Nod. Z+  >P60 Nod. Z+ vs P12-17 Nod. Z+  >P60 Floc. Z+ vs P18-29 Floc. Z+ | | 0.14  0.40  <0.0001  0.82 | | 62.2 ± 2.5 Hz vs 56.4 ± 2.8 Hz  62.2 ± 2.5 Hz vs 58.4 ± 2.9 Hz  62.2 ± 2.5 Hz vs 24.9 ± 3.8 Hz  67.3 ± 9.8 Hz vs 64.7 ± 14.2 Hz | | 48 cells vs 32 cells from 62 mice  48 cells vs 20 cells from 54 mice  48 cells vs 7 cells from 41 mice  9 cells vs 3 cells from 11 mice | |
| Supplement Figure2.1 C_1_  Simple spike CV (*in vitro*) | | Kruskal-Wallis test  multiple comparisons | | | P3-P5 LIII vs P3-P5 LX  P6-P9 LIII vs P6-P9 LX  P10-P11 LIII vs P10-P11 LX  P12-P17 LIII vs P12-P17 LX  P18-P29 LIII vs P18-P29 LX  P30-P59 LIII vs P30-P59 LX  >P60 LIII vs >P60 LX | | 0.13  0.007  0.13  0.10  0.007  0.003  <0.0001 | | 0.24 ± 0.03 vs 0.44 ± 0.06  0.43 ± 0.21 vs 0.34 ± 0.08  0.10 ± 0.01 vs 0.09 ± 0.01  0.08 ± 0.01 vs 0.09 ± 0.04  0.14 ± 0.01 vs 0.10 ± 0.01  0.14 ± 0.04 vs 0.09 ± 0.01  0.13 ± 0.01 vs 0.08 ± 0.01 | | 23 cells vs 18 cells from 3 mice  30 cells vs 25 cells from 6 mice  37 cells vs 36 cells from 3 mice  23 cells vs 25 cells from 4 mice  43 cells vs 73 cells from 9 mice  44 cells vs 50 cells from 7 mice  102 cells vs 121 cells from 17 mice | |

| **Related Figure** | **Test applied** | | **Groups** | | ***p*-value** | | **Descriptive values** | **Number of cells / mice** | |  |
| --- | --- | --- | --- | --- | --- | --- | --- | --- | --- | --- |
| Supplement Figure2.1 C_2_  Simple spike regularity index (*in vitro*) | Kruskal-Wallis test  multiple comparisons | | P3-P5 LIII vs P3-P5 LX  P6-P9 LIII vs P6-P9 LX  P10-P11 LIII vs P10-P11 LX  P12-P17 LIII vs P12-P17 LX  P18-P29 LIII vs P18-P29 LX  P30-P59 LIII vs P30-P59 LX  >P60 LIII vs >P60 LX | | 0.67  0.008  0.33  0.017  <0.0001  <0.0001  <0.0001 | | 0.006 ± 0.002 vs 0.002 ± 0.001  0.032 ± 0.005 vs 0.010 ± 0.003  0.044 ± 0.005 vs 0.055 ± 0.007  0.037 ± 0.003 vs 0.064 ± 0.006  0.016 ± 0.002 vs 0.060 ± 0.004  0.026 ± 0.004 vs 0.086 ± 0.009  0.017 ± 0.001 vs 0.082 ± 0.004 | 23 cells vs 18 cells from 3 mice  30 cells vs 25 cells from 6 mice  37 cells vs 36 cells from 3 mice  23 cells vs 25 cells from 4 mice  43 cells vs 73 cells from 9 mice  44 cells vs 50 cells from 7 mice  102 cells vs 121 cells from 17 mice | |  |
| Supplement Figure2.2 A_1_  Simple spike rate *in vivo* and *in vitro* | Kruskal-Wallis test  multiple comparisons | | P12-P17 LIII vs P12-P17 Z–  P18-P29 LIII vs P18-P29 Z–  P30-P59 LIII vs P30-P59 Z–  >P60 LIII vs >P60 Z–  P12-P17 LX vs P12-P17 Z+  P18-P29 LX vs P18-P29 Z+  P30-P59 LX vs P30-P59 Z+  >P60 LX vs >P60 Z+ | | 0.40  0.034  <0.001  <0.0001  0.18  0.001  <0.001  <0.0001 | | 51.7 ± 3.4 Hz vs 57.3 ± 5.3 Hz  55.8 ± 5.6 Hz vs 69.0 ± 4.4 Hz  59.1 ± 4.8 Hz vs 78.4 ± 3.2 Hz  61.2 ± 2.5 Hz vs 89.2 ± 2.3 Hz  34.8 ± 2.0 Hz vs 42.3 ± 5.7 Hz  42.4 ± 2.9 Hz vs 55.9 ± 3.2 Hz  45.6 ± 3.6 Hz vs 56.7 ± 2.2 Hz  46.3 ± 1.9 Hz vs 61.5 ± 2.4 Hz | 23 cells vs 20 cells  43 cells vs 17 cells  44 cells vs 43 cells  102 cells vs 70 cells  24 cells vs 25 cells  34 cells vs 73 cells  47 cells vs 50 cells  62 cells vs 121 cells | |  |
| Supplement Figure6.1 A_1_  OKR gain | Mixed-effects repeated measures | | Young vs Adult | | 0.84 | | 0.60 ± 0.09 vs 0.59 ± 0.10 | 16 mice vs 11 mice | |  |
| Supplement Figure6.1 A_2_  VOR gain | Mixed-effects repeated measures | | Young vs Adult | | <0.0001 | | 0.42 ± 0.09 vs 0.61 ± 0.09 | 16 mice vs 11 mice | |  |
| Supplement Figure6.1 A_3_  VVOR gain | Mixed-effects repeated measures | | Young vs Adult | | 0.009 | | 0.89 ± 0.01 vs 0.96 ± 0.01 | 16 mice vs 11 mice | |  |
| Supplement Figure6.1 B_1_  OKR Phase | Mixed-effects repeated measures | | Young vs Adult | | 0.96 | | -15.5 ± 6.7 ° vs -15.4 ± 5.0 ° | 16 mice vs 11 mice | |  |
| Supplement Figure6.1 B_2_  VOR phase | Mixed-effects repeated measures | | Young vs Adult | | 0.002 | | 29.4 ± 5.8 ° vs 21.4 ± 4.5 ° | 16 mice vs 11 mice | |  |
| Supplement Figure6.1 B_3_  VVOR phase | Mixed-effects repeated measures | | Young vs Adult | | 0.97 | | 1.33 ± 0.46 ° vs 1.35 ± 0.13 ° | 16 mice vs 11 mice | |  |
|  |  |  | |  | |  | | |  | |

| **Related Figure** | **Test applied** | | | **Groups** | | | ***p*-value** | | | **Descriptive values** | | | **Number of cells / mice** | | |
| --- | --- | --- | --- | --- | --- | --- | --- | --- | --- | --- | --- | --- | --- | --- | --- |
| Supplement Figure6.2 B  VOR phase reversal gain | | | Mixed-effects repeated measures | | | Young vs Adult day1  Young vs Adult day2  Young vs Adult day3  Young vs Adult day4  Young vs Adult day5 | | | <0.0001  <0.0001  0.002  0.54  0.38 | | | 0.28 ± 0.04 vs 0.52 ± 0.04  0.11 ± 0.01 vs 0.33 ± 0.03  0.15 ± 0.02 vs 0.22 ± 0.02  0.21 ± 0.01 vs 0.20 ± 0.01  0.23 ± 0.007 vs 0.21 ± 0.005 | | | 16 mice vs 11 mice |
| Supplement Figure6.2 C  VOR gain consolidation | | One-way ANOVA  multiple comparisons | | | Young vs Adult day1-2  Young vs Adult day2-3  Young vs Adult day3-4 | | | 0.0005  0.0006  0.007 | | | 101.8 ± 7.0 % vs 62.6 ± 4.6 %  71.7 ± 9.0 % vs 33.0 ± 7.7 %  85.4 ± 5.4 % vs 55.6 ± 9.2 % | | | 16 mice vs 11 mice | |
| Supplement Figure6.2 D  VOR phase reversal gain training | | Mixed-effects repeated measures | | | Young vs Adult day1  Young vs Adult day2  Young vs Adult day3  Young vs Adult day4  Young vs Adult day5 | | | <0.0001  0.01  0.0003  0.26  0.22 | | | 0.11 ± 0.02 vs 0.16 ± 0.02  0.81 ± 0.05 vs 0.66 ± 0.06  0.70 ± 0.02 vs 0.59 ± 0.02  0.78 ± 0.01 vs 0.74 ± 0.02  0.82 ± 0.01 vs 0.79 ± 0.01 | | | 16 mice vs 11 mice | |
| Supplement Figure6.2 E  VOR phase reversal phase training | | Mixed-effects repeated measures | | | Young vs Adult day1  Young vs Adult day2  Young vs Adult day3  Young vs Adult day4  Young vs Adult day5 | | | 0.97  <0.0001  <0.0001  <0.0001  <0.0001 | | | 45.8 ± 1.11 ° vs 45.8 ± 1.03 °  174.8 ± 2.74 ° vs 155.1 ± 3.68 °  180.3 ± 0.74 ° vs 172.5 ± 1.38 °  180.7 ± 0.54 ° vs 174.0 ± 0.77 °  181.1 ± 0.18 ° vs 175.7 ± 0.71 ° | | | 16 mice vs 11 mice | |
| Supplement Figure6.4 B_1_  Unconditioned response onset | | Mixed-effects repeated measures | | | Young vs Adult | | | 0.41 | | | 5.61 ± 0.15 msec vs 6.41 ± 0.10 msec | | | 18 mice vs 13 mice | |
| Supplement Figure6.4 B_2_  Unconditioned response peaktime | | Mixed-effects repeated measures | | | Young vs Adult | | | 0.55 | | | 67.2 ± 1.0 msec vs 69.0 ± 1.0 msec | | | 18 mice vs 13 mice | |
